# The Prevalence of Distress and Depression among Women in Rural Sichuan Province

**DOI:** 10.1371/journal.pone.0161097

**Published:** 2016-08-15

**Authors:** Peiyuan Qiu, Eric D. Caine, Fengsu Hou, Catherine Cerulli, Marsha N. Wittink, Jin Li

**Affiliations:** 1 West China School of Public Health, Sichuan University, Chengdu, China; 2 Department of Psychiatry, University of Rochester Medical Center, Rochester, NY, United States of America; 3 School of Public Health, Sun Yat-sen University, Guangzhou, China; 4 Sun Yat-sen Global Health Institute, Institute of State Governance, Sun Yat-sen University, Guangzhou, China; 5 Department of Psychiatry, West China Hospital, Sichuan University, Chengdu, China; Iranian Institute for Health Sciences Research, ISLAMIC REPUBLIC OF IRAN

## Abstract

**Background:**

In this paper, we report findings regarding the prevalence of expressed distress and depressive conditions among women living in a rural region of Sichuan Province. As well, we know of no data among women in rural China that examine whether “depression,” as categorically defined in classifications such as the DSM, adequately captures the expressed distress and symptomatic complaints of women in rural China.

**Methods:**

A multistage sampling method was employed to recruit the target population. The Center for Epidemiologic Studies Depression Scale (CES-D) was used to measure distress symptoms, and MINI International Neuropsychiatric Interview (MINI) was employed to determine the prevalence of diagnosable depression and other disorders.

**Results:**

Among 1898 rural women, 12.4% (236) scored ≥16 on the CES-D, indicative of distress, and a subset of 7.7% (146) had scores ≥21, highly suggestive of a clinically significant disorder. We found that 49.8% women with ≥16 CES-D score were identified as showing features consistent with a current major depressive episode (MDE) vs. 1.9% in a sample of randomly selected women with <16 CES-D score. Among respondents, 30 of 84 (35.7%) scoring 16–20 and 83 of 143 (58.0%) scoring ≥21 reported symptoms consistent with MDE. 25.1% of women with a positive CES-D score did not describe symptoms consistent with any DSM-IV disorder.

**Conclusions:**

We found a higher portion of women reporting significant distress than previously described. Among them, there was a clear gradient, such that 41.7% of women with moderate distress did not have a psychiatric diagnosis, and even among those with more severe symptoms, 15.4% did not manifest a DSM-specific psychiatric condition.

## Introduction

In this paper we report findings regarding the prevalence of expressed distress and depressive conditions among women living is a rural region of Sichuan Province. Women in rural China continue to serve as primary care givers for their families and more often are “left behind” as their spouses seek employment in urban centers and industrial zones. Even as overall suicide rates apparently have been declining during recent years, those of younger and middle aged rural women continue to remain close to those of men [1].

Rural areas in China, especially those distant from major metropolitan centers, remain less modernized and less influenced by western medical concepts. The education level in rural China is lower and it is likely that rural residents are more likely to retain traditional notions of mental health and related interventions. Now that the PRC government has committed the country to modernizing health services across rural regions of China, as well as providing enhanced medical insurance coverage, it essential to better understand the burden of mental distress and clinically definable conditions.

Similar to Western nations [1–11], the reported rate of major depressive disorder in China among women is higher than that found among men (2.60% vs. 1.55%), and the rate of depression in rural regions (2.24%) is higher than that in urban areas [9]. However, we know of no data among women in rural China that examine whether “depression,” as categorically defined in classifications such as the DSM, adequately captures the expressed distress and symptomatic complaints of women in rural China. As well, many people and families are embarrassed by the presence of mental health concerns [12–15], and avoid expressing distress in a clinically meaningful fashion.

In this paper, part of a series arising from this research, we describe the prevalence of symptoms of distress using the Center for Epidemiologic Studies Depression Scale (CES-D) among women, ages 16 years and older, in a rural region of Sichuan Province. We also determine among those “cases,” the prevalence of diagnosable “clinical conditions,” including depressive disorders and other conditions by the MINI International Neuropsychiatric Interview (MINI).

## Methods

For this study we focused on the rural region of Guangyuan City, which lies in the northern, mountainous area of Sichuan Province. In China, “cities” stretch beyond central urban areas and often encompass distinctly rural communities. Guangyuan City includes three districts, and four counties, with a population of 2.48 million– 820,000 (33.1%) urban and 1.66 million (66.9%) rural. The average annual net 2010 income for rural residents was ¥5,649 (approximately $911) and the average living expenses were ¥4,406 (approximately $711) [16].

### Sampling

We sought to include a representative sample of women, ages 16 years and older, living in the rural Lizhou district of Guangyuan City. To ensure study participants represented a range of different socio-economic sectors, we used a multistage sampling method: We divided the nine towns in Lizhou District into three levels—low, middle, and high-income. From each income level, one town was randomly selected as our research site, and within each town, three to five villages were selected randomly. We used the method of random number table by SPSS to select the towns and villages. Thirteen villages were recruited in the study. We then used the Chinese household registration system (*hukou*) to identify eligible women living in the villages. In total, we identified 5865 possible participants. All of the women, ages 16 years and older, currently living in the 13 villages were eligible. We excluded—i.e., did not approach to obtain consent—women if they had a history of severe mental illness or cognitive impairment based on reports from family members or village doctors, or if after providing consent, they chose to discontinue interviews (thus, not completing the stusy protocol).

### Recruitment

The study was conducted during the summer of 2012. Local government and health departments played an important role in the recruitment of participants, a necessary aspect of conducting studies in rural areas of China. With the help of the village leaders and village doctors, we first organized public information sessions about the research project. Interviewers were graduate and undergraduate medical students enrolled at Sichuan University who had been trained in survey and semi-structured interviewing techniques. The interviewers spent three to four days in each village. In the villages where there was more population density, we worked with the village leaders, doctors, and seniors to walk door-to-door and conduct our survey; they served the essential role of introducing interviewers to potential participants. In the villages where the population density was lower, local residents with motorcycles drove the interviewers to do survey door-to-door. In one town where the villages perched on a mountainside, we were not able to complete a full door-to-door survey due to heavy summer rains during the study period. All participants were given a token of appreciation for their time consisting of toiletry items (such as toothpaste and soap) worth ¥5 (about $0.80). Interviews lasted 20–70 minutes. When an eligible participant was unavailable or not home, we returned twice before defining them as unavailable.

**Fig 1:**
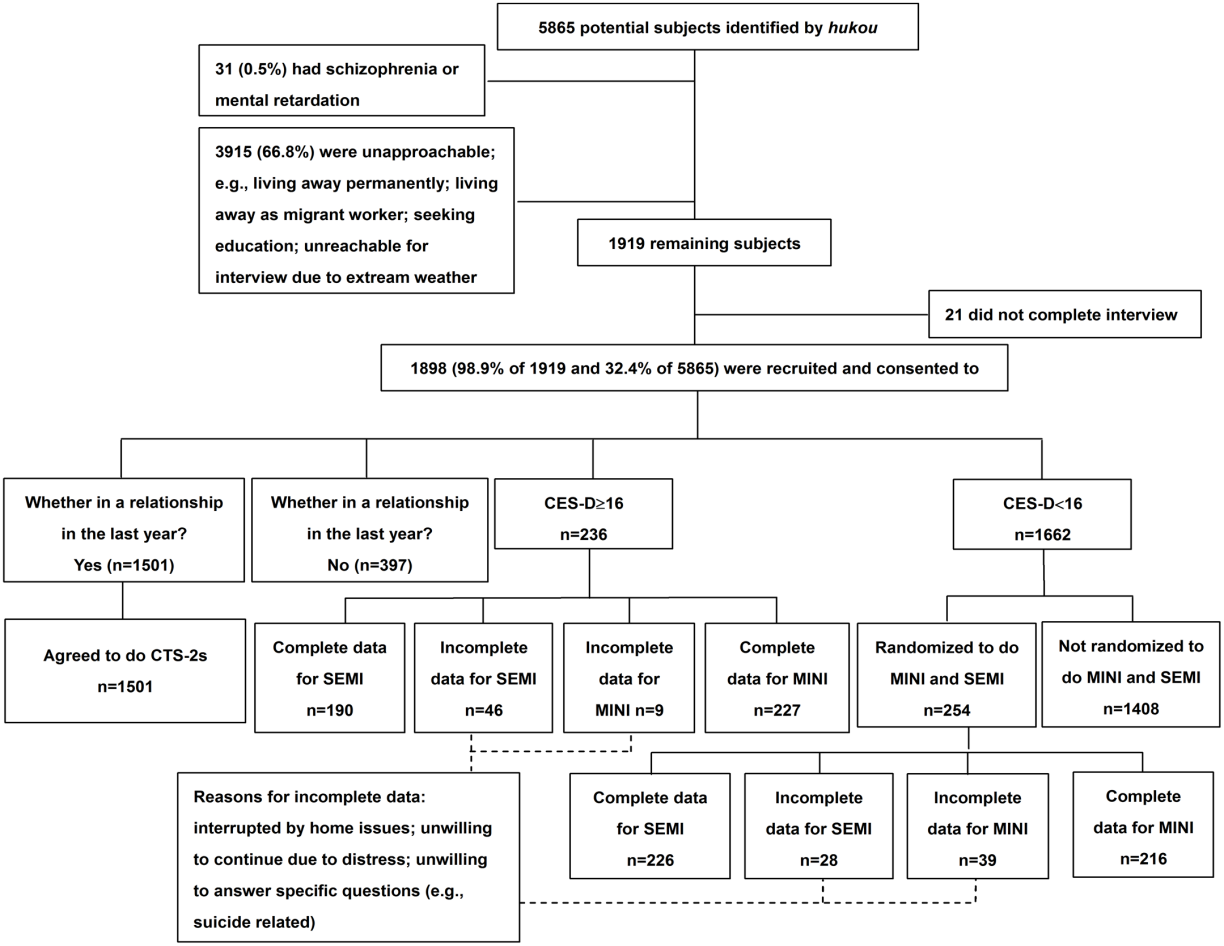
Recruitment of subjects.

The protocol including verbal the informed consent was reviewed and approved by the Medical Ethics Committee of Sichuan University (2011004). The University of Rochester Research Subjects Review Broad reviewed and approved analyses of de-identified data. In the study, we recruited women aged 16 years and older—the designated age for adult consent in China. The Medical Ethics Committee of Sichuan University approved the consent procedure for participants younger than 18 years. We asked all participants to provide verbal informed consent, as many of the rural women could not write. The interview process also required affirmative assent to answer our inquires; as seen in Fig 1, 21 participants discontinued the basic interview, which constituted a removal of affirmative consent and the protocol was discontinued with none of their data included in analyses. The study was deemed to pose a low risk for participants, and was conducted in accordance with the Declaration of Helsinki.

### Interviewer Training and Quality Control

A total of 24 interviewers were selected from undergraduate and postgraduate medical students from West China Medical Center, Sichuan University. All interviewers completed a structured training program. The training program involved: one day training to introduce the topic of affective disorders; two days training on administrating MINI; and two days training on administrating computer based interviews, interview skills, response skills, and research ethics. (Interviewers also participated in training sessions related to other interview tools, which will be discussed in subsequent related publications.) We first demonstrated standardized interviews with two experienced graduate students. Interviewers next paired to conduct mock interviews under supervision. Each interviewer also conducted a practice interview in front of the whole team.

During the field study, the 24 interviewers were divided into three groups. Each group was led and supervised by an experienced field researcher. The interviewers checked the participant responses immediately after the interviews were conducted. If any items were missing or inconsistent information was found, the interviewer would reassess the participant regarding these items. At the end of each interview day, the three group supervisors checked all interview protocols. In addition, the team leader conducted quality control meetings for the entire team every evening to discuss any difficulties or challenges encountered, and to develop consensus strategies to address these issues.

### Protocol and Measures

All participants completed socio-demographic questions including age, marital status, living situation, education, occupation, ethnic background, religion, and household income. In addition, all participants completed questions regarding self-rated health, diagnosed chronic diseases, and health care utilization, based on items from the National Health Services Survey [17]. For this aspect of the study, all participants completed the CES-D [18]. The CES-D, a self-report inventory, contains 20 items that are scored by respondents to indicate the frequency of symptoms during the previous week, using a scale of 0 –less than a day, 1–1–2 days, 2–2–3 days, and 3–5–7 days. It has a very wide spectrum of noted symptoms, which makes it not simply a depression scale, but also a distress scale. A score of 16 or more is indicating the likely presence of distress. (Of note, past epidemiological research in China has demonstrated the validity and reliability of the CES-D, and similar to other countries, found that a score of 16 was applicable[19–22]).

Those with a CES-D score of ≥16 were further assessed for categorically defined mental disorders using the Mini-International Neuropsychiatric Interview (MINI) and interviewed for their perceptions regarding distress and depression [23, 24]. A sample of lower scoring participants also completed the MINI. The MINI is a short, structured diagnostic interview to explore 17 disorders according to Diagnostic and Statistical Manual (DSM)-III-R diagnostic criteria, designed to allow administration by non-specialized interviewers. The MINI contains 120 questions and screens 17 axis I DSM-III-R disorders for 24 current and lifetime diagnoses. In our results, we reported current diagnoses. Beside, in our study, given that alcohol and drug abuses, anorexia nervosa, and bulimia nervosa in rural women are rare, we did not include them. As well, since women with psychosis were not eligible for the study, psychotic disorders were not investigated.

### Analysis

We applied Chi-square test and t-test to analyze differences of demographic factors between people with CES-D score ≥16 and < 16. Furthermore, we employed bivariate logistic regression to define demographic factors associated with depressive symptoms.

We categorized participants into four groups based on age: 16–24 years old, 25–44 years old, 45–64 years old, and ≥ 65 years old. We divided participants into five education groups: lack of formal schooling (no school), primary level of education (≤6 years), junior high school level of education (7–9 years), high school level of education and above (≥10 years). Occupational status was categorized into five groups: agriculture work, non-agriculture work, homemakers, students, and unemployed. In addition, we categorized subjects into three groups based on their annual household income: ≤ 10,000, 10,000–20,000, and >20,000. Living status was categorized into three groups: living alone, living with core family and living with extended family (including living with relatives and friends). We dichotomized marital status into two groups: married and not married, dichotomized ethnic background into Han ethnic and others, and dichotomized religious into two groups: without any religious and with any kind of religious. In our study, statistical significance was established at *p* < 0.05.

## Results

We recruited with verbal consent 1919 women in the study; 1898 of them completed the interview. The CES-D scores ranged from 0 to 57 (average = 7.2±8.4; median = 5). Among the participants, 1662 (87.6% of 1898) respondents scored below 16 (<16), while 236 (12.4%) scored 16 or above (≥16). Of the total sample, 146 (7.7%) had scores ≥ 21, highly suggestive of a clinically significant depressive condition; [18] that is, one that is associated with functional deficits as well as reported symptoms. Table 1 shows the distribution of participants by age, marital status, religion, living situation, education attainment, occupation, and household income. The results of the bivariate logistic regression (Table 2) indicated that women who were poor and unemployed were more likely to have depressive symptoms. Religious impact on depressive symptoms was marginal.

**Table 1:** Sociodemographics Characteristics of Women.

	Total	CES-D ≥ 16	CES-D < 16	Chi-square
	N (%)	N (%)	N (%)	T-test
	n = 1898	n = 236	n = 1662	
**Total**				-
**Participant Characteristics**				
**Age**				0.010
16–24	143(7.5)	11(4.7)	132(7.9)	
25–44	605(31.9)	61(25.8)	544(32.7)	
45–64	843(44.4)	127(53.8)	716(43.1)	
65-	307(16.2)	37(15.7)	270(16.2)	
**Marital Status**				0.176
Single	342(18.0)	50(21.2)	292(17.6)	
Married	1556(82.0)	186(78.8)	1370(82.4)	
**Ethnic Background**				0.119
Han	1893(99.7)	234(99.2)	1659(99.8)	
Others	5(0.3)	2(0.8)	3(0.2)	
**Religion**				0.020
Yes	88(4.6)	18(7.6)	70(4.2)	
No	1810(95.4)	218(92.4)	1592(95.8)	
**Living Situation**				0.483
Alone	75(4.0)	10(4.3)	65(3.9)	
With Core Family	680(35.9)	76(32.3)	604(36.4)	
With extended family (including living with relatives and friends)	1141(60.1)	149(63.4)	992(59.7)	
**Education Attainment**				0.029
No School	724(38.1)	102(43.2)	622(37.4)	
Less than 6 Years	706(37.2)	91(38.6)	615(37.0)	
7 to 9 Years	324(17.1)	35(14.8)	289(17.4)	
10 and above	144(7.6)	8(3.4)	136(8.2)	
**Occupation**				0.001
Agricultural work	1117(58.9)	146(61.9)	971(58.5)	
Non-agricultural work	234(12.3)	20(8.5)	214(12.9)	
Homemakers	326(17.2)	34(14.4)	292(17.6)	
Students	43(2.3)	1(0.4)	42(2.5)	
Unemployed	177(9.3)	35(14.8)	142(8.5)	
**Household Income**				0.010
≤ 10000	685(36.1)	103(43.6)	582(35.0)	
10000–20000	483(25.4)	62(26.3)	421(25.3)	
> 20000	730(38.5)	71(30.1)	659(39.7)	

**Table 2:** Bivariate logistic regression—demographic factors and depressive symptoms.

Participants’s Characteristics	OR(95% CI)	*P*
**Income**	0.82(0.69–0.96)	0.014
**Education**	0.91(0.76–1.08)	0.27
**Occupation**		
Agricultural work	—	—
Non-agricultural work	0.74(0.45–1.24)	0.26
Homemakers	0.84(0.56–1.25)	0.38
Students	0.19(0.03–1.44)	0.11
Unemployed	1.66(1.10–2.50)	0.02
**Religious**		
No religious	—	—
Any religious	1.74(1.00–3.01)	0.05

Among the women scoring <16 (n = 1662), we randomly selected and consented 254 for further study. Compared to the larger asymptomatic group, there were no significant differences in educational attainment, marital status, and household income, although we did find modest differences in age and occupation distribution. Asymptomatic women who were randomized to do MINI had a lower average age (44.4 vs 48.9 years for “positive” responders) and were more likely to be involved in agricultural work (66.2% vs 57.3%).

Two hundred twenty-seven (227) of the 236 participants scoring ≥16 on the CES-D completed the MINI to determine the prevalence of diagnosable depression and other conditions; as well, 216 of 254 of our random sample of participants with a CES-D <16 completed the protocol (Fig 1).

Table 3 shows that 113 of the 227 (49.8%) women with ≥16 CES-D score were identified as showing features consistent with a current major depressive episode (MDE) vs. four of the 216 (1.9%) in the comparison group. Of those individuals with scores of 16–20, 30 of 84 (35.7%) reported symptoms consistent with MDE; of those with CES-D scores ≥21, 83 of 143 (58.0%) also had consistent features. Of those scoring ≥16, 24 of 227 (10.6%) presented with symptoms of dysthymia in contrast to one (0.5%) among the non-distressed women. For those with scores of 16–20, 5 of 84 (6.0%) apparently suffered dysthymia, while for those ≥21, 19 of 143 (13.3%) had a similar presentation. Thus, among women with a positive CES-D score, 57 of 227 (25.1%) did not describe conditions that were diagnosed in a fashion consistent with a DSM-IV disorder, 35 of 84 (41.7%) for those scoring 16–20 and 22 of 143 (15.4%) of those ≥21.

**Table 3:** MINI Diagnosed Conditions among Participants with Distress.

		CES-D < 16 N (%) n = 216	CES-D ≥ 16 N (%) n = 227		
			CES-D = 16–20	CES-D ≥ 21	CES-D ≥ 16
			N (%)	N (%)	N (%)
			n = 84	n = 143	n = 227
**Primary Diagnosis**	Major Depressive Episode (MDE)	4 (1.9)	30(35.7)	83(58.0)	113(49.8)
	Dysthymia	1(0.5)	5(6.0)	19(13.3)	24(10.6)
	Bipolar Disorder^a^	2(0.9)	0(0.0)	4(2.8)	4(1.8)
	Manic/Hypomanic Episode	3(1.4)	2(2.4)	2(1.4)	4(1.8)
	Anxiety Disorders^b^	11(5.1)	12(14.3)	13(9.1)	25(11.0)
**No Diagnosis**		195(88.9)	35(41.7)	22(15.4)	57(25.1)

^a.^ MINI does not have a diagnosis of bipolar disorder. In our sample, if respondent had major depressive episode and manic/hypomanic episode, she was classified as bipolar disorder in our study.

^b.^ Anxiety disorders include panic disorder, agoraphobia, social phobia, obsessive-compulsive disorder, posttraumatic stress, and generalized anxiety disorder. As long as respondents had one of these anxiety disorders, they were counted as having anxiety disorders.

## Discussion

We found a higher prevalence than previously reported of personal distress and major depression in these rural communities [9, 25, 26], which are bearing the burden of heavy rural-to-urban migration. Among the unapproachable potential subjects listed in the village *hukou*, the vast majority were migrant workers who lived at distant locations. Compared to those remaining in the villages, they were younger, and likely healthier and better educated. Our results suggest that, among the women who did remain in rural regions during this period of rapid urbanization in China, there are many who are especially vulnerable to significant clinical conditions. Moreover, we would anticipate that as urbanization accelerates further, there will be an increasing tendency to draw younger, more mobile, economically prepared women to the cities, further concentrating more vulnerable, less educated, older and less employable individuals, posing future challenges. We are mindful, for example, that problems such as suicide among elders have not declined in rural regions in the same fashion as the decline among younger individuals [1], and such rates may tend to increase during years ahead as elders’ children and extended familes continue to migrate away.

More than three decades ago, Kleinman argued that “depression” in China was manifest in a distinctive, culturally congruent fashion [2, 10], and that the Western notions of psychopathology were inadequate for capturing persons’ presentations. Anthropological and psychiatric research indicated in years past that Chinese people often expressed distress arising from interpersonal or social situations by way of complaints of anxiousness, headaches, insomnia, chest discomfort, and dizziness, rather than using “depression” to characterize their condition. This was captured by the term neurasthenia; except for post-influenza neurasthenia, this concept largely was dropped from the Western diagnostic nosology with the promulgation of DSM-III in 1980[8, 10, 27, 28]. By 2000, the diagnostic use of neurasthenia also had virtually disappeared among Chinese psychiatrists; its replacement, depression, has become the new term for medical professionals, and increasingly, for patients themselves [29]. Studies in Hong Kong [4] and in modern urban centers in China [5, 7] document that the Western construct of depressive disorders now is deeply anchored in those settings and has apparent clinical utility. Moreover, epidemiological studies now undertaken in China use the DSM, Western-based nosology as their frame of reference[9].

Our study found that 137 (60.4%) women with a ≥16 CES-D score reported symptoms indicative of a MINI diagnosed mood disorders—either MDE or dysthymic disorder, while 33 (14.6%) had other primary diagnoses. However, 57 (25.1%) did not report symptoms indicative of any specific diagnosis. Even among women with a CES-D score ≥21, 22 (15.4%) did not have any diagnosis, reinforcing the finding that highly symptomatic persons may not report clinical features consistent with a DSM diagnosis. In the comparison group of women with a CES-D score <16, only five of 216 (2.4%) received a MINI diagnosis of MDE or dysthymic disorder. Overall, 195 (88.9%) had no diagnosis.

These results suggested that there were many moderately and severely distressed women who did not present their complaints in a fashion consistent with DSM categorical conditions. Clearly this dissonance diminished with increasing severity; yet even at higher CES-D levels, we found many who were not coded diagnostically. This is potentially relevant when considering future service needs, and also when planning how best to train village and town doctors to recognize persons in distress.

The CES-D has been used across nations and populations with reported variation in its prediction of diagnosed clinical conditions. A study of evaluating reliability and validity of CES-D in Greek populaion showed that sensitivity and specificity of CES-D were 92.5% and 85.0%, respectively, if 21 was used as the cutoff score [30]. Another involving older Chinese showed that 22 was the optimum threshold in its sample, with sensitivity, specificity, positive predictive value (PPV) and negative predictive value (NPV) were 0.75, 0.51, 0.55 and 0.72 [31]. Various studies from the 1990s, using a a CES-D score of 16, found widely varying sensitivity (from 63.3% to 100.0%) and specificity (from 53.0% to 93.9%) [32, 33]. These results are consistent with our finding that there are many significantly distressed individuals whose conditions do not neatly fit into categorical diagnoses.

Our findings point to the presence of meaningful and important levels of personal distress that are not captured in a categorical fashion. At the same time, a majority of the participants’ presentations were consistent with DSM-based patterns of psychopathology. Put another way, we partly confirmed Kleinman’s past observations using other methods—that is, to a modest degree—even as the bulk of our findings at this time point to the potential utility of categorical psychiatric descriptions in these rural villages. Whether the differences in the prevalence of distress versus depression specifically reflect the factors posited by Kleinman remains to be determined.

We caution regarding several potential limitations. The study was conducted in one rural area of Sichuan Province. In light of China’s extraordinary diversity, we must be careful generalizing our results to other rural regions. In addition, we are very aware that because of internal migration in China, we could not approach many women who were registered in study villages, potentially compromising the representativeness of our sample. And women who migrate to city may have different characteristics from those who are left-behind. However, using *hukou* as a method to identify eligible women living in rural villages such as these remains the best choice currently. Finally, we did not use psychiatrists to administer the MINI, thus requiring caution when considering the accuracy of our psychiatric diagnoses.

## Conclusion

Women with clinically significant depressive symptoms were older, less educated, involved in farming or unemployed, and economically disadvantaged. Overall, 25% of women at or above the CES-D threshold had no diagnosis. We saw in our results a clear gradient, such that 41.7% of women with moderate distress did not have a psychiatric diagnosis, and even among those with more severe symptoms, 15.4% did not manifest a DSM-specific psychiatric condition. Distress and depression are relatively common among women residing in these rural communities where many people have left for urban opportunities. These conditions pose significant personal and community challenges, and point to pressing needs in China for developing basic, community-focused mental health services.

## Supporting Information

S1 Data Set This is the dataset. (SAV)
